# Period prevalence of self-reported headache in the general population in Germany from 1995–2005 and 2009: results from annual nationwide population-based cross-sectional surveys

**DOI:** 10.1186/1129-2377-14-11

**Published:** 2013-02-14

**Authors:** Andreas Straube, Bernhard Aicher, Steffanie Förderreuther, Thomas Eggert, Janin Köppel, Stefan Möller, Roland Schneider, Gunther Haag

**Affiliations:** 1 Deptment Neurology, Ludwig-Maximilians-Universität München, Klinikum Großhadern, Munich, Germany; 2 Boehringer Ingelheim Pharma GmbH & Co. KG, Ingelheim am Rhein, Germany; 3 Ipsos Operations GmbH, Mölln, Germany; 4 Michael-Balint-Klinik, Königsfeld im Schwarzwald, Germany

**Keywords:** Headache, Prevalence, General population, Age, Gender, Epidemiology, Income, Education

## Abstract

**Background:**

Although primary headache is the most frequent neurological disorder and there is some evidence that the prevalence rates have increased in recent years, no long-term data on the annual prevalence of headache are available for Germany. The objective of the study was therefore to obtain long-term data on the period prevalence of headache in the general population in Germany by means of population-based cross-sectional annual surveys (1995–2005 and 2009).

**Methods:**

These surveys were conducted as face-to-face paper-and-pencil interviews from 1995 through 2004, and from 2005 onwards as computer-aided personal interviews. The reported headaches were self-diagnosed by the interviewees. Per year, approximately 640 trained interviewers interviewed between 10,898 and 12,538 German-speaking individuals aged 14 and older and living in private households in the whole of Germany (response rate: 67.4% and 73.1%, gross samples: 16,026 to 18,176 subjects). A total of more than 146,000 face-to-face interviews were analyzed.

**Results:**

The one-year headache prevalence remained stable over the entry period, with 58.9% (95%CI 57.7–60.1) to 62.5% (95%CI 61.3–63.7) (p=0.07). Women showed consistently higher prevalence rates than men (females: 67.3 (95%CI 65.7–68.9) to 70.7% (95%CI 69.1–72.3), males: 48.4% (95%CI 46.5–50.3) to 54.3% (95%CI 52.4–56.2)), and both sexes showed a bell-shaped age dependence with peaks in the 30–39 age group. A stable slightly higher prevalence was observed in urban versus rural areas (p<0.0001), and there was also a significant trend towards higher prevalence rates in groups with a monthly household income larger than 3,500 € (p=0.03).

**Conclusion:**

The overall headache prevalence remained stable in Germany in the last 15 years.

## Background

Headache constitutes a major public health problem in many countries [1]. The global prevalence for current headaches of the adult population is 46%; the life-time prevalence is 66% [2]. In Asia, Australia, Europe, and North America, the headache prevalence in the general population is approximately 50%; in Africa it is distinctly lower at 20% [2,3]. The reason for this difference is not known. For Europe a meta-analysis reviewed the 49 available surveys of headache prevalence, 34 of which cover the occurrence of headache within one year or less, defined as “current headache” [3], with a prevalence of 53% on average [3]. The different epidemiological studies differ depending on the interview technique used (face-to-face interview, telephone interview, questionnaire mailings), the questionnaires, and the inclusion and exclusion criteria for the interview, the profession of the interviewer, the interviewed age groups, and the representativeness of the interviewed group for the general population. Therefore, it is not surprising that the different surveys for a particular country show prevalence rates that differ considerably from one another [3]. For Germany, two cross-sectional surveys on the prevalence of headaches in the general population are available [4,5]. The German Migraine and Headache Society (DMKG) Headache Study investigated the regional differences in headache prevalence in Germany and therefore could not generate prevalence data for the general population [4]. Such regional differences in headache prevalence within one country have also been described, for example, in the Austrian Self-Reported Morbidity (SERMO) Study [6] for Austria, or for the prevalence of migraine in Spain [7]. The German National Telephone Health Interview Survey (GNT-HIS) 2004 [5] reported the headache prevalence for the general population [8]. The one-year prevalence for 2004 is 60.2%, with only slight regional variations between 59.1% and 61.5% [5].

All these surveys are cross-sectional surveys, collecting the prevalence within a period (in most studies 1, 6, or 12 months) or as life-time prevalence only once at a certain time point. Therefore they are not useful to answer the question of whether the headache prevalence rates increase or remain stable over time. Only a limited number of cross-sectional surveys determine headache prevalence in a longitudinal study with repeated surveys. The Nord-Trøndelag Health Studies HUNT 2 and HUNT 3 were done at intervals of eleven years [9], and the American Migraine I and II and AMPP studies at roughly 10-year intervals [10-12].

The objective of the present study was therefore to obtain long-term data (from 1995 to 2009) on the prevalence of self-reported headaches in the general population in Germany by a population-based cross-sectional survey.

## Methods

### Study design

These cross-sectional surveys were conducted as face-to-face multi-topic interviews, quarterly in three waves in the months of March, June, September, and November/December from 1995 through 2009. Between 1995 and 2004, the interviews were conducted with paper and pencil (PAPI), whereas from 2005 onwards, interviews were carried out via computer-aided personal interviews (CAPI). No changes were made to the questionnaire during the period described.

The interview years 2006 to 2008 are not part of this analysis due to major methodological differences (further details on headache disease and medication used were recorded), which could have influenced the response rate and could possibly have led to doubts concerning the comparability of data.

Per survey year, around 640 trained interviewers carried out the field work (research institute: Ipsos GmbH (Mölln/Germany)). Thus, one interviewer did 19 interviews on average per year, referring to the gross sample. One complete interview took about 50 minutes. In the course of the multi-topic survey, each interviewee was asked questions on six topics. The interviewees did not receive any incentive.

### Interviewees

The interviewees were German-speaking individuals aged 14 years and older living in private households in Germany. Based on the ADM’s (Arbeitsgemeinschaft Deutscher Marktforschungsinstitute) random networks, each year a population-based multi-layer random sample was drawn that included between 10,898 and 12,538 net interviewees (gross sample 16,026 to 18,176). The sample was drawn in three steps and was representative of the German-speaking population living in private households:

1. The basis for the sample is a pool of ADM networks for details see [13]. Per survey wave, an average of 200 sample points was determined at random. The ADM-Sample is an area sample. The frame consists of about 53,000 areas. Communal statistics and data from navigation systems are used to create the areas. Each area/point comprises on average 700 private households. In addition, several attributes (town size, statistical code etc.) are assigned to the points. This allows a regional stratification of the points.

2. Participating households were selected using the random-route method. Starting from a randomly selected address the interviewer determines the target households according to strictly defined rules. Description of random route rule: from selected address, go left, then take the first possible right, go right and then take the first possible left. The interviewer has to count the households from left to right. Every 5th household is the target household for the interview.

3. The person in the household with the closest birthday was selected for the interview [14,15]. The household was contacted up to three times in order to conduct the interview with the target person.

All interviewees gave their informed consent and the surveys were done under the German law for market polls.

### Structural weighting of the sample

The three-stage ADM sample system leads to equal chances at a household level, but not at the level of household members. This makes a household transformation necessary: a multiplication of all data sets by the number of household members and a subsequent division by the average household size. In this way, the sample is transformed into a sample on the basis of household members. Besides the described transformation, no further weighting procedure is carried out.

### Questionnaire

The following socio-demographic data were collected: state, population of the home town/area, sex, age, education, current occupation, and net household income. The interviewees also reported on their illnesses and diseases during the past twelve months. In addition to headaches, the questionnaire scanned e.g. for menstrual cramps, shoulder and neck tension, constipation, common cold or cough. Twenty-three to thirty-one diseases and conditions were queried in a random order. This multi-topic procedure contributed at least partly to the blinding of the topic of special interest among the interviewees and the interviewers. The headache diseases were recorded using terms normally used by headache patients themselves to describe their condition. The questions to identify headache were: “Now you see a list of illnesses and diseases on the screen (CAPI: questionnaire): headache due to shoulder and neck tension, migraine, tension-type headache, or headache caused by weather changes. Which of these did you have in the last 12 months? Please tick the box (yes, no, don’t know/not applicable).”

### Quality assurance

Quality assurance of the interviews followed DIN ISO 20252 [16]. This standard of the German Institute for Standardization has been the valid international quality standard for market research, opinion polls, and social research since 2006. This DIN standard includes requirements for quality assurance in market and social research institutions, including the outsourcing of services to external vendors; for the study design, including the necessary cooperation with the sponsor; for the gathering of data in quantitative and qualitative surveys, including the recruitment and training of interviewers; for data processing and data management; and for the reporting to the sponsor. Accordingly, the institute asked at least 10% of the interviewees for a short feedback after the interview, either in writing or via telephone, to check whether the interview had actually taken place, how long it had taken, and what topics had been covered.

### Analysis

For evaluation, the gathered data was categorized, including differentiation between interviewees from former East Germany and from former West Germany. Since 2005, 15 years after the reunification of Germany, only Germany has been recorded. The reason for this was that the economic and political differences between the former East and former West Germany had decreased dramatically over the years. Thus, no further differences in lifestyle could be observed between the different regions. The cut-off point between rural and urban populations was a population r of 50,000 inhabitants. Net household incomes were divided into four categories: (a) up to 999€, (b) 1,000€ - 2,499€, (c) 2,500€ - 3,499€, (d) 3,500€ and more per month. Current occupations were also divided into four categories: (a) self-employed and freelancers, (b) public officers, skilled and managing employees, (c) unskilled and semi-skilled workers as well as the temporarily unemployed, (d) trainees, non-working persons and retirees.

### Statistical evaluation

The prevalence of self-reported headaches was determined as the quotient of the number of people out of the net sample who reported having had headaches during the period of twelve months prior to the interview and the total number of people out of the net sample, including the 95% confidence interval (95% CI).

The effects of the factors year, state, region, education, and income on the prevalence of headache were tested with chi-square tests on k-by-2 contingency tables, where k denotes the level of the particular factor. Additionally, for the factor year a trend analysis was performed according to Bortz et al. [17]. This test provides the alpha-probability for the null hypothesis that the frequency of headache does not increase or decrease linearly over the years.

## Results

**Figure 1:**
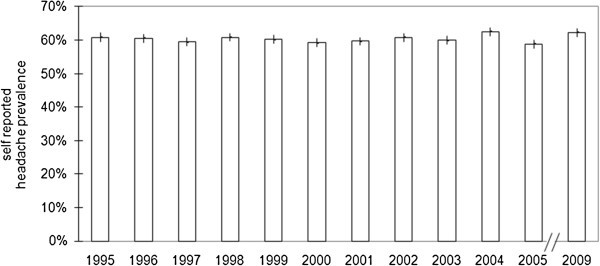
**One-year headache prevalence rates (at 95% confidence intervals) in Germany in the years 1995–2005 and 2009.** There was no significant change in the headache prevalence over the years (p=0.07). Basic population is the German-speaking population aged 14 years and older.

**Figure 2:**
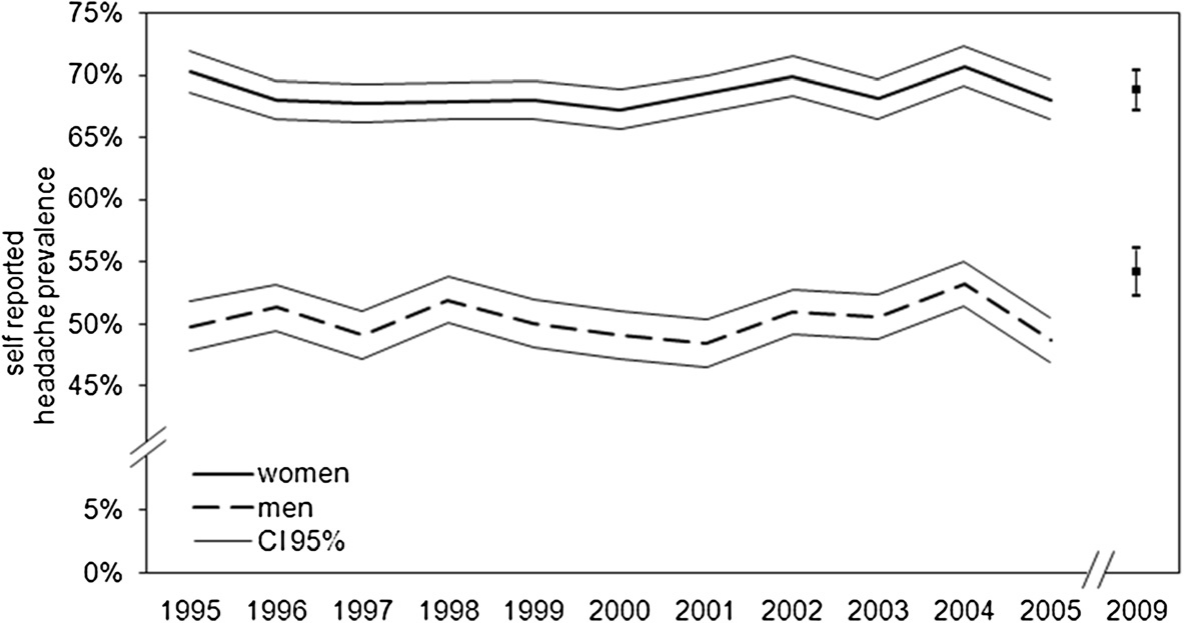
**One-year headache prevalence rates (at 95% confidence intervals) by gender 1995–2005, 2009.** Basic population is the German-speaking population aged 14 years and older.

The gross sample sizes ranged between 16,026 and 18,176 people, the response rates of the interviews between 1995 and 2009 ranged between 67.4% and 73.1% (Table 1) (a total of 146,253 face-to-face interviews were included in the analysis). The prevalence of self-reported headaches showed a not significant slight tendency to increase with time (trend: z=1.8 p=0.07), ranging between 58.9 and 62.5% (Figure 1). The sex-specific differences remained stable, too, with 67.3 to 70.7% of women reporting headaches in the last 12 months compared to 48.4 to 54.3% of men. For men, the range of variation (5.9%) was almost twice as high as that for women (3.4%) (Table 1; Figure 2).

**Table 1:** Results of the annual cross-sectional surveys of period prevalence rates of self-reported headaches in Germany from 1995–2005 and 2009

**Year of survey**		**1995**	**1996**	**1997**	**1998**	**1999**	**2000**	**2001**	**2002**	**2003**	**2004**	**2005**	**2009**
**General population in million inhabitants (≥ 14 years)**		**62.73**	**62.97**	**63.12**	**63.24**	**63.51**	**63.78**	**63.83**	**64.1**	**64.25**	**64.43**	**64.72**	**64.87**
**Total**	n =	10898	12239	12389	12067	12447	12503	12538	12351	12487	12064	12150	12120
	**HP**	**60.9%**	**60.6%**	**59.5%**	**60.9%**	**60.2%**	**59.3%**	**59.7%**	**60.8%**	**60.0%**	**62.5%**	**58.9%**	**62.3%**
	CI 95%	59.6 - 62.2	59.4 – 61.8	58.3 - 60.7	59.7 - 62.1	59.0 - 61.4	58.1 - 60.5	58.5 - 60.9	59.6 - 62.0	58.8 - 61.2	61.3 - 63.7	57.7 – 60.1	61.1 - 63.5
**Women**	n =	5915	6817	6908	6737	7047	6999	7037	6433	6691	6453	6386	6676
	**HP**	**70.2%**	**68.0%**	**67.8%**	**67.9%**	**68.0%**	**67.3%**	**68.5%**	**69.9%**	**68.1%**	**70.7%**	**68.0%**	**68.9%**
	CI 95%	68.5 - 71.9	66.5 - 69.5	66.3 - 69.3	66.4 - 69.4	66.5 - 69.5	65.7 - 68.9	67.0 - 70.0	68.3 - 71.5	66.5 - 69.7	69.1 - 72.3	66.4 - 69.6	67.3 - 70.5
**Men**	n =	4984	5423	5482	5330	5401	5505	5501	5917	5796	5609	5765	5444
	**HP**	**49.8%**	**51.3%**	**49.1%**	**51.9%**	**50.1%**	**49.1%**	**48.4%**	**50.9%**	**50.5%**	**53.2%**	**48.7%**	**54.3%**
	CI 95%	47.8 - 51.8	49.4 - 53.2	47.2 - 51.0	50.0 - 53.8	48.2 - 52.0	47.2 - 51.0	46.5 - 50.3	49.1 - 52.7	48.7 - 52.3	51.4 - 55.0	46.9 - 50.5	52.4 - 56.2

General population of Germany (older than 14 years and German-speaking), gross sample, and response rates of the IPSOS surveys; the table also shows the one-year prevalence rates of self-reported headaches at 95% confidence intervals (CI95%) for the general total sample as well as for the following sub-samples: men/women. (HP: headache prevalence in %; CI: confidence interval).

The one-year headache prevalence of the former East and West Germany showed no significant differences (60.2% to 60.5%) (Table 2) (contingency table χ^2^(1)= 0.78; p=0.38). Interestingly, the one-year headache prevalence was consistently slightly lower in rural areas (population < 50,000) than in urban regions (population > 50,000) (Table 2) (rural: 59.9%; urban: 61.3%) (contingency table χ^2^( (1)= 29.67; p<0.0001).

**Table 2:** Results of the annual cross-sectional surveys of prevalence rates of self-reported headaches in Germany from 1995-2005 and 2009

**Year of survey**		**1995**	**1996**	**1997**	**1998**	**1999**	**2000**	**2001**	**2002**	**2003**	**2004**	**2005**	**2009**
**General population in million inhabitants (≥ 14 years)**		**62.73**	**62.97**	**63.12**	**63.24**	**63.51**	**63.78**	**63.83**	**64.1**	**64.25**	**64.43**	**64.72**	**64.87**
**East Germany**	n =	2709	2677	2994	3041	2804	2631	2770	2629	2659	2485	2863	2571
	**HP**	**61.4%**	**60.8%**	**57.5%**	**61.2%**	**60.3%**	**58.3%**	**63.2%**	**63.8%**	**59.1%**	**58.5%**	**56.3%**	**62.8%**
	CI 95%	58.8 - 64.0	58.2 - 63.4	55.0 - 60.0	58.7 - 63.7	57.7 - 62.9	55.7 - 60.9	60.7 - 65.7	61.2 - 66.4	56.5 - 61.7	55.8 - 61.2	53.7 - 58.9	60.2 - 65.4
**West Germany**	n =	8190	9563	9396	9026	9643	9873	9768	9721	9828	9579	9288	9549
	**HP**	**60.7%**	**60.5%**	**60.2%**	**60.8%**	**60.2%**	**59.5%**	**58.7%**	**60.0%**	**60.2%**	**63.6%**	**59.6%**	**62.2%**
	CI 95%	59.2 - 62.2	59.1 - 61.9	58.8 - 61.6	59.4 - 62.2	58.8 - 61.6	58.1 - 60.9	57.3 - 60.1	58.6 - 61.4	58.8 - 61.6	62.2 - 65.0	58.2 - 61.0	60.8 - 63.6
**Rural areas with ≤ 50,000 inhabitants**	n =	6170	7035	7307	6991	7063	7226	7424	7591	5943	7810	7955	7557
	**HP**	**60.5%**	**60.1%**	**60.9%**	**61.2%**	**60.2%**	**57.2%**	**59.6%**	**58.4%**	**58.5%**	**61.2%**	**58.2%**	**62.2%**
	CI 95%	58.8 - 62.2	58.5 - 61.7	59.3 - 62.5	59.6 - 62.8	58.6 - 61.8	55.6 - 58.8	58.0 - 61.2	56.8 - 60.0	56.7 - 60.3	59.7 - 62.7	56.7 - 59.7	60.6 - 63.8
**Urban areas with > 50,000 inhabitants**	n =	4729	5202	5082	5076	5385	5277	5114	4759	6544	4253	4196	4562
	**HP**	**61.4%**	**61.3%**	**57.5%**	**60.4%**	**60.2%**	**62.1%**	**59.8%**	**64.6%**	**61.3%**	**64.9%**	**60.0%**	**62.5%**
	CI 95%	59.4 - 63.4	59.4 - 63.2	55.6 - 59.4	58.5 - 62.3	58.3 - 62.1	60.2 - 64.0	57.9 - 61.7	62.7 - 66.5	59.6 - 63.0	62.9 - 66.9	57.9 - 62.1	60.5 - 64.5

The table shows the one-year prevalence rates of self-reported headaches at 95% confidence intervals (CI95%) for residents of former East and West Germany (East: 60.2%, West: 60.5%, contingency table χ^2^(1) = 0.78; p=0.38), residents of cities of 50,000 inhabitants and more, and residents of places up to 49,999 inhabitants ( Rural: 59.9%; Urban: 61.3% contingency table χ^2^( (1)= 29.67; p<0.0001). Basic population is the German-speaking population aged 14 years and older. (HP: headache prevalence in %; CI: confidence interval).

The level of education and, consequently, also the monthly income seems to affect lifestyle in some way and therefore also probably affects headache prevalence (Table 3). The headache prevalence for the group of “self-employed and freelancers” (62.6%) is slightly lower than the prevalence for the group of “skilled and managing employees” (63.9%) and that is slightly lower than the prevalence in the group of “unskilled and semiskilled workers” (65.1%) (Table 3). Overall, the one-year headache prevalence for the group of “public officers and skilled employees” even increased slightly from 1998 onwards. The lower prevalence (55.8%) in the group of “trainees, non-working and retirees” can partly be explained by the generally lower one-year headache prevalence in the younger and older age groups which were more prevalent in this group (see below). The differences in prevalence rates between the groups were significant with P values from p< 0.02 to p< 0.001.

**Table 3:** Results of the annual cross-sectional surveys of prevalence rates of self-reported headaches in Germany from 1995–2005 and 2009

**Year of survey**		**1995**	**1996**	**1997**	**1998**	**1999**	**2000**	**2001**	**2002**	**2003**	**2004**	**2005**	**2009**
**General population n million inhabitants (≥ 14 years)**		**62.73**	**62.97**	**63.12**	**63.24**	**63.51**	**63.78**	**63.83**	**64.1**	**64.25**	**64.43**	**64.72**	**64.87**
**Self-employed, freelancers**	n =	622	736	728	715	709	696	706	703	753	730	732	668
	**HP**	**64.6%**	**59.1%**	**65.4%**	**61.6%**	**62.8%**	**65.3%**	**59.7%**	**61.6%**	**64.7%**	**64.5%**	**56.7%**	**65.3%**
	CI 95%	59.3 - 69.9	54.1 - 64.1	60.5 - 70.3	56.5 - 66.7	57.8 - 67.8	60.3 - 70.3	54.6 - 64.8	56.5 - 66.7	59.9 - 69.5	59.6 - 69.4	51.6 - 61.8	60.2 - 70.4
**Public officers, skilled and managing employees**	n =	4633	5109	4984	4757	5019	4936	4945	4698	4461	4488	4684	5017
	**HP**	**61.6%**	**63.2%**	**61.3%**	**62.9%**	**63.4%**	**62.7%**	**63.5%**	**64.7%**	**65.3%**	**67.5%**	**64.2%**	**66.5%**
	CI 95%	59.6 - 63.6	61.3 - 65.1	59.4 - 63.2	61.0 - 64.8	61.5 - 65.3	60.8 - 64.6	61.6 - 65.4	62.8 - 66.6	63.3 - 67.3	65.5 - 69.5	62.3 - 66.1	64.6 - 68.4
**Unskilled and semi-skilled workers**	n =	1123	1315	1487	1500	1490	1340	1481	1335	1503	1392	1291	1378
	**HP**	**65.2%**	**65.3%**	**63.3%**	**66.4%**	**64.7%**	**64.1%**	**66.7%**	**68.2%**	**62.6%**	**68.1%**	**61.6%**	**64.9%**
	CI 95%	61.2 - 69.2	61.6 - 69.0	59.9 - 66.7	63.0 - 69.8	61.3 - 68.1	60.5 - 67.7	63.3 - 70.1	64.7 - 71.7	59.2 - 66.0	64.7 - 71.5	57.8 - 65.4	61.3 - 68.5
**Trainees, non-working persons (e.g. house-wives), retirees**	n =	4521	5078	5186	5045	5230	5511	5406	5614	5766	5413	5362	4950
	**HP**	**58.6%**	**57.1%**	**55.8%**	**57.2%**	**55.6%**	**54.1%**	**54.3%**	**55.7%**	**54.5%**	**56.8%**	**53.9%**	**57.1%**
	CI 95%	56.6 - 60.6	55.2 - 59.0	53.9 - 57.7	55.3 - 59.1	53.7 - 57.5	52.2 - 56.0	52.4 - 56.2	53.9 - 57.5	52.7 - 56.3	54.9 - 58.7	52.0 - 55.8	55.1 - 59.1
**Household income (net) per month ≤ 999 €**	n =	1210	1309	1343	1299	1294	1268	1121	1318	2216	1027	1037	874
	**HP**	**61.4%**	**61.2%**	**60.4%**	**59.8%**	**60.6%**	**58.3%**	**59.5%**	**60.6%**	**60.5%**	**61.1%**	**55.5%**	**62.1%**
	CI 95%	57.5 - 65.3	57.4 - 65.0	56.7 - 64.1	56.0 - 63.6	56.8 - 64.4	54.5 - 62.1	55.4 - 63.6	56.9 - 64.3	57.6 - 63.4	56.9 - 65.3	51.2 - 59.8	57.5 - 66.7
**Household income (net) per month 1,000€ - 2,499 €**	n =	6947	7693	7923	7834	7894	7785	7712	7662	7431	7085	7379	7197
	**HP**	**61.0%**	**60.9%**	**59.1%**	**61.4%**	**60.1%**	**58.4%**	**59.7%**	**60.8%**	**59.5%**	**61.9%**	**58.3%**	**60.2%**
	CI 95%	59.4 - 62.6	59.4 - 62.4	57.6 - 60.6	59.9 - 62.9	58.6 - 61.6	56.9 - 59.9	58.2 - 61.2	59.2 - 62.4	57.9 - 61.1	60.3 - 63.5	56.7 - 59.9	58.6 - 61.8
**Household income (net) per month 2,500€ - 3,499 €**	n =	1837	2096	2057	1993	2201	2407	2459	2256	1844	2598	2729	2932
	**HP**	**60.7%**	**59.6%**	**60.7%**	**61.9%**	**60.5%**	**62.0%**	**59.4%**	**60.1%**	**60.8%**	**63.1%**	**60.0%**	**66.4%**
	CI 95%	57.5 - 63.9	56.6 - 62.6	57.7 - 63.7	58.9 - 64.9	57.6 - 63.4	59.2 - 64.8	56.7 - 62.1	57.2 - 63.0	57.6 - 64.0	60.5 - 65.7	57.4 - 62.6	64.0 - 68.8
**Household income (net) per month ≥ 3,500 €**	n =	586	686	589	656	601	724	763	700	651	1115	979	1102
	**HP**	**60.0%**	**58.3%**	**63.3%**	**59.6%**	**63.4%**	**64.8%**	**60.9%**	**63.1%**	**61.2%**	**65.7%**	**63.1%**	**65.2%**
	CI 95%	54.4 - 65.6	53.1 - 63.5	57.8 - 68.8	54.3 - 64.9	58.0 - 68.8	59.9 - 69.7	56.0 - 65.8	58.1 - 68.1	55.9 - 66.5	61.7 - 69.7	58.9 - 67.3	61.2 - 69.2

The table shows the one-year prevalence incomes. Basic population is the German-speaking population aged 14 years and older. HP: headache prevalence. (HP: headache prevalence in %; CI: confidence interval)rates of self-reported headaches at 95% confidence intervals (CI95%) for a) different occupational groups, and b) the strata for the different net household.

If we take a look at the one-year headache prevalence in relation to the monthly net household income, the highest income group shows the highest range of variation of up to 7.4% in the headache prevalence since 1998 (Table 3). Overall the variation in the prevalence between the different income groups was smaller than between the educational groups (< 999€: 60.1%; 1000–2499€: 60.1%; 2500–3499€: 61.4%; >3500€: 62.7%). No differences in the one-year headache prevalence can be found for the groups with the lower income (p=0.96); in contrast the differences in prevalence between the two groups with the higher income and the groups with lower income were significant (P values between p=0.009 and p<0.001) (Table 3).

**Figure 3:**
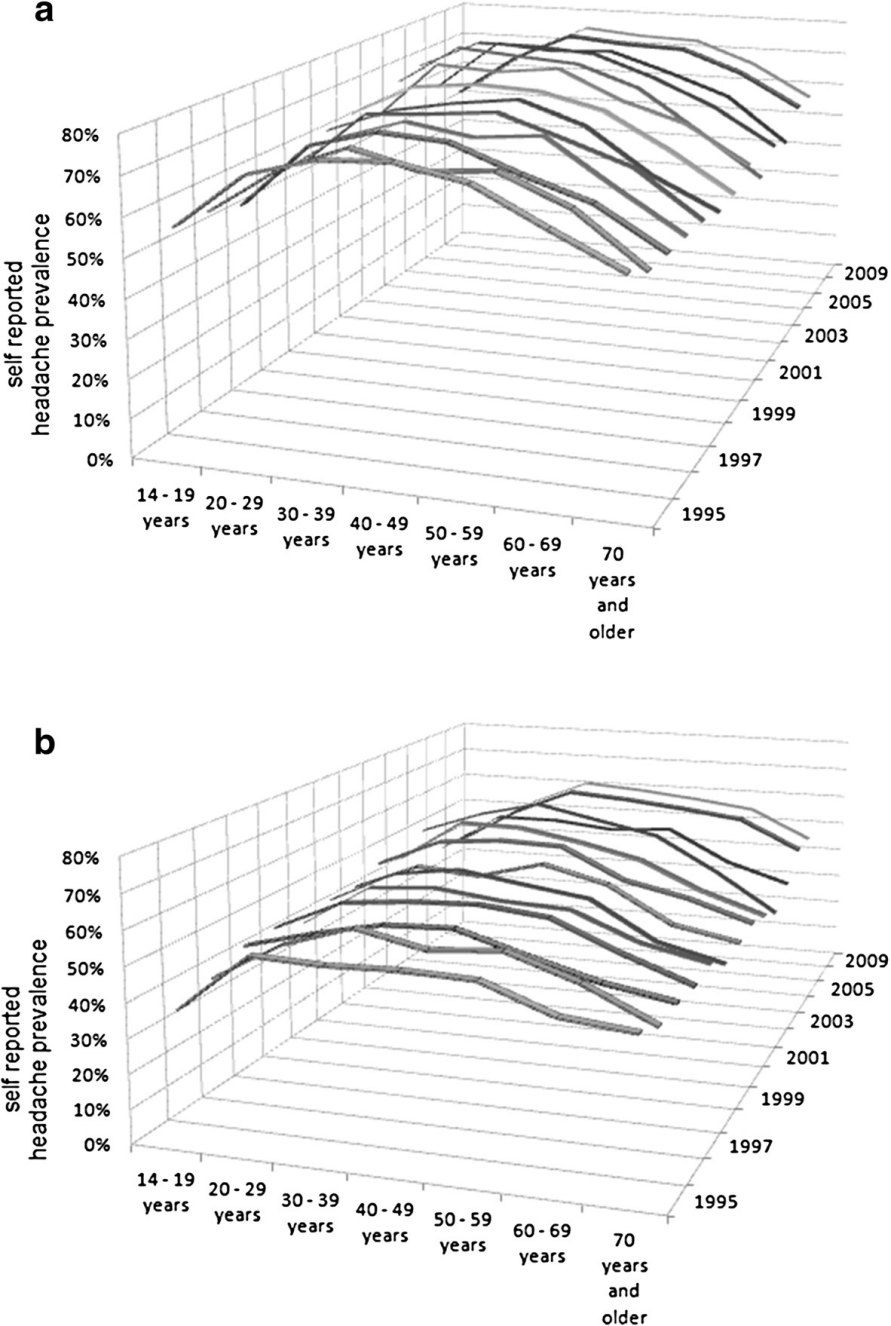
**One-year headache prevalence rates of age cohorts of the annual cross-sectional surveys of period prevalence rates of self-reported headaches in Germany from 1995–2005 and 2009: a) Women; b) Men.** Basic population is the German-speaking population aged 14 years and older.

From 1995 to 2005 and in 2009 a typical dependence of the headache prevalence on age could be demonstrated in the German population. The youngest age group (14 to 19 years) and the two older age groups (60 and older) consistently show lower prevalence than those with an age of 20–59 years. Although these values vary slightly over the years, the bell-shaped age distribution does not change during the investigated time period (Figure 3). This age distribution even remains, if we look at women (Figure 3a) and men (Figure 3b) separately.

## Discussion

The number of longitudinal epidemiological studies on the period prevalence rates of headaches is limited. Our study is the first which determines the one-year prevalence of self-reported headache in representative samples of the German population over a long time period of 15 years by using the same methods (annual face to face interview). The most important result is that from 1995 to 2009 the one-year headache prevalence in the German general population is stable at 58.9 to 62.5%. The German National Telephone Health Interview Survey 2004 (GNTHIS), which accessed a large representative sample of the general adult population in Germany aged 18 years and older (n = 7,341), determined the one-year prevalence of headaches for the year 2004 as 60.3% (66.6% for women, 53.0% for men) [5]. Our study showed prevalence rates of 62.5% (70.7% for women, 53.2% for men) in 2004 (Table 1). The German DMKG Headache Study with 7,417 interviewees, analyzing three cross-sectional surveys conducted in three German regions (Augsburg, Dortmund, Pomerania) between 2002 and 2004, found a sex- and age-stratified, pooled 6-month prevalence of 49.5% [4]. The investigated regions in that study, however, are not representative of the general population in Germany [4]. Another survey which used mailed questionnaires (4,061 interviewees) and a market research household panel, found a lifetime prevalence for headaches of 71.4% [18]. In their meta-analysis “Global Burden of Headache”, Stovner et al. – based on 107 surveys – report 47% as the global period prevalence for “current headache”, which included 1 year, 3 months and ”time not stated”. When limited to surveys among adults, they report a prevalence of 46% [2]. Thus, the one-year headache prevalence for Germany between 1995 and 2009 is about 10% to 15% higher. The reason for that is not clear, especially since the prevalence of migraine was slightly lower in the DMKG study than in comparable studies from other European countries [1,4]. Only three other European surveys investigated the one-year prevalence and were conducted as face-to-face interviews, as in our survey. For Austria with 997 interviewees, the one-year prevalence was 49.4% (women 54.6%, men 43.6%) [19]; in Finland with 200 interviewees, it was 77% (women 83%, men 69%) [20]; and in Georgia with 1,145 interviewees, it was 46.3% [21].

An important result of our study was that the one-year prevalence of general headaches remains stable over a period of 15 years in Germany. This fact is quite remarkable since in this period dramatic political and economic changes took place in East Germany, where – beginning in 1990 – a socialistic economic system was transformed into a capitalist economic system. For the population of the former GDR this change in the economic and social system was equal to a loss of secure employment and confidence. It is obvious that such changes in lifestyle can have an influence on the stress experienced, which probably is one of the reasons for headache. Surprisingly we did not see such an effect in our survey which may indicate that the majority of the interviewees did not feel such a stress.

These stable prevalence rates are in agreement with other longitudinal studies from different countries. Four large cross-sectional surveys in the US also show stable one-year prevalence of migraine over a period of about ten years (1989 to 1998) [11,12,22-25]. Two methodically identical surveys conducted in France at an interval of ten years also showed a stable prevalence for migraine too [26,27]. Also, the Norwegian HUNT 2 and HUNT 3 studies do not show any change in the one-year prevalence rates within eleven years [9,28]. The results from the GNTHIS 2004 survey support our finding that there is no difference in the headache prevalence between East and West Germany either [5].

Another interesting observation in our study is that the prevalence rates were slightly, but noticeably lower in rural/small-town regions than in urban areas with over 50,000 inhabitants, which is in agreement with results from Austria [19]. In contrast, the 1989 survey in the USA shows only minor regional differences – only mountainous areas, which are in general rural, showed higher one-year prevalence rates which is in contrast to our results. But here only interviewees with self-defined severe migraine were included and the regions were much larger than in Germany and Austria [29]. The size of the place of residence had only a minor impact in this US survey with a slightly higher prevalence for severe migraine in the rural regions (< 50,000 inhabitants). A Spanish study also reported a slightly higher prevalence of general pain conditions (including headache) in urban areas compared to rural regions [30].

The question of whether household income and social situation have an influence on the headache prevalence rates is controversial. An English survey showed somewhat higher 3-month headache prevalence for interviewees from socially higher, non-manual working population strata than for manual workers [31]. But this difference may be explained by differences in the age and sex distribution in the interviewed groups [31]. In contrast a Spanish survey showed that the one-year prevalence of migraine was higher for subjects doing housework and unemployed subjects than for working people [32]. But this study made no adjustment for the presumably higher percentage of women among those doing housework and the different age distributions. Although various surveys, in particular those from the US, associate the prevalence of headache diseases and especially of migraine with lower household income, the results are also contradictory. In the American Migraine Study II, migraine prevalence increased continuously with decreasing household income and was highest for men and women with an annual income below $15,000 [11]. This confirmed results from 1989, which were gathered from a panel of 15,000 households [12] – for both migraine as well as all severe headaches. An analysis of the data of the National Health Interview Survey of 1989 also showed that self-reported migraine was slightly more common among those with a household income of less than $10,000 compared to those with a higher household income in both men and women [33]. In the US ARIC study, a household income of less than $16,000 vs. $75,000 and more was associated with a slightly increased prevalence for migraine with and without aura, but not with other headache types [34]. In contrast, in a survey from Philadelphia County, which shows demographic similarities with the general US population, no association between income and migraine prevalence was found; neither for the crude nor for the age-adjusted prevalence [25]. In a recent study again an increased headache frequency in the group with the lowest annual income (<$22,500) was reported [35]. Pryse-Phillips et al., in a Canadian survey on migraine and tension-type headache, could otherwise not find any correlation between these headache types and household income [36]. In contrast to most of the studies from the USA, in our study the headache period prevalence was constantly slightly higher at a monthly income of 2,500€ or more than with lower monthly income. Two other German surveys also show a higher migraine period prevalence in the medium and higher socio-economic groups [5,6]. The reasons for these different results remain unclear, but it should be taken into consideration that the social systems in Europe and USA are quite different.

The prevalence show a distinct dependence on sex and age, which results in a stable, almost identical bell-shaped age distribution over all investigated years. Women always showed higher one-year prevalence rates than men in all age groups. These dependencies have been described repeatedly for migraine [1,2,37], for headaches in general, however, less data is available. Since tension-type headaches are more frequent than migraine in all the samples, the reported age dependence indicates that it is also applicable to tension-type headache and not only to migraine. This corresponds with the results from regional studies (Baltimore County in USA, Nord-Trondelag County in Norway) [38,39]. On the other hand, a nationwide representative study in France showed period prevalence of non-migraine headaches to be age- or sex-dependent only for women younger than 25 years [26]. Boardman et al. discuss possible reasons for this age-dependent decrease in period prevalence of headaches, for example a possible recall bias due to the increase of other more relevant diseases among the elderly [31]. Also, Russell et al. ask whether the age-dependent decrease, which is statistically significant in their survey only for men, but not for women, is a real phenomenon or caused by recall bias [40]. However, a 15-year cohort study with employees of a French gas and power supply company showed a clear decrease of the prevalence of headaches at the point of retirement of the employees, with the decrease being particularly distinct in the group of employees with high work-related stress [41].

The interpretation of the results should take the strengths and limitations of this study into account. The choice of interview method depends essentially on the contents of the information to be gathered [42]. Much more complex information can be collected in face-to-face interviews than by mail surveys [42]. Telephone interviews tend to be much shorter, e.g. in a Spanish telephone survey on pain, the interview lasted 10.5 minutes on average [30], while it took about 90 minutes in the French GRIM2000 face-to-face migraine survey [28] and 50 minutes in our study. Face-to-face interviews achieve the highest response rates and are terminated early by the interviewees less often than telephone surveys or mail surveys [43,44]. In our study, the response rate of 67.4% to 73.1% was higher than that of the DMKG survey with 66.9% [4] and about as high as in a regional Croatian face-to-face survey [45]. It was also higher than that of surveys with other data collection methods, such as that of 53.3% in the GNTHIS 2004 (telephone interviews) [5] or that of 62% in a US telephone survey [23], or 37.1% in a Spanish telephone survey [7]. The most important aspect of our study is the large number of interviewees with more than 12,000 interviews per year over a total of 15 years, i.e. a database of more than 146,000 analyzed face-to-face interviews. Furthermore, the multi-topic survey contributed at least partly to the required “blinding” of the topic and helped to minimize a recall bias [46]. The method used ensured that there was a standardized interview situation over the whole period [43,44]. One of the limitations under discussion in some surveys is the lack of ICHD-II classification of the headaches and that the interviews were not done by headache experts. Nevertheless, Rasmussen points out that a physician might subconsciously influence patient responses into patterns corresponding to his ideas of IHS headache classification [47]. Even with adequate training and the use of structured interviews, misclassification cannot be excluded completely and an underreporting of migraine might still be possible. As this survey only asked about the presence of any kind of headache, independent of its type, intensity, frequency, and duration, the use of trained interviewers is not a relevant limitation in our survey. Moreover, this kind of question and the restriction to one year could minimize recall bias, a serious problem of retrospective surveys. The study was not designed to evaluate risk factors for headache and we therefore did not apply multivariate analyses.

## Conclusion

In summary, this large-scale longitudinal survey over a period of almost 15 years does not indicate any general increase in the one-year prevalence of self-reported headaches in Germany. Women show continuously higher prevalence rates than men, and both women and men exhibit some age dependence, although the topic was headaches in general and not just migraine. There are only minor regional differences and there is no difference between former East and West Germany. A relatively stable difference between rural and urban regions, however, is remarkable, as well as a trend towards higher prevalence rates among people with higher to high income.

## Competing interests

AS received honoraria for participation in clinical trials, contribution to advisory boards or oral presentations from: Allergan, Berlin Chemie, Boehringer Ingelheim, Desitin, MSD Germany, Pfizer, St. Jude, and scientific grants from the German Research Council, the BMBF, and the Kröner-Fresenius-Foundation. BA, JK, and RS are employees of Boehringer Ingelheim Pharma GmbH & Co. KG, Germany. SM is an employee of IPSOS Operations GmbH, Mölln, Germany. SF received honoraria for contribution to advisory boards or oral presentations from Allergan, Boehringer Ingelheim and MSD. GH received honoraria for participation in clinical trials, contribution to advisory boards or oral presentations from: Allergan, Berlin-Chemie, Boehringer Ingelheim, Glaxo Smith Kline, Janssen Cilag, and MSD. TE reported no conflict of interest.

## Authors’ contributions

AS wrote the final manuscript and performed the statistical analysis; BA initiated the study and wrote the first draft; SF and GH contributed to the discussion of the results and to the final manuscript; TE provided statistics; JK, SM, and RS were part of the steering committee and helped in the ascertainment of the data. All authors read and approved the final manuscript.

## References

[B1] Stovner LJ Zwart JA Hagen K Terwindt GM Pascual J Epidemiology of headache in Europe Eur J Neurol 2006 14 333 345 10.1111/j.1468-1331.2006.01184.x 16643310

[B2] Stovner LJ Hagen K Jensen R The global burden of headache:A documentation of headache prevalence and disability worldwide Cephalalgia 2007 14 193 210 10.1111/j.1468-2982.2007.01288.x 17381554

[B3] Stovner LJ Andree C Prevalence of headache in Europe: a review for the Eurolight project J Headache Pain 2010 14 289 299 10.1007/s10194-010-0217-0 20473702 PMC2917556

[B4] Pfaffenrath V Fendrich K Vennemann M Meisinger C Ladwig K-H Evers S Straube A Hoffmann W Berger K Regional variations in the prevalence of migraine and tension-type headache applying the new IHS criteria: the German DMKG Headache Study Cephalalgia 2009 14 48 57 10.1111/j.1468-2982.2008.01699.x 18771491

[B5] Radtke A Neuhauser H Prevalence and burden of headache and migraine in Germany Headache 2009 14 79 89 10.1111/j.1526-4610.2008.01263.x 19125877

[B6] Rieder A Lobentanz I Zeitlhofer J Mitsche N Lawrence K Schwarz B Kunze M Background morbidity of headache in an adult general population - Results of the Austrian SERMO (Self-Reported Morbidity) Study Wien Klin Wochenschr 2004 14 176 181 10.1007/BF03040484 15088992

[B7] Matias-Guiu J, Porta-Etessam J, Mateos V, Diaz-Insa S, Lopez-Gil A, Fernandez C on behalf of the scientific committee of the PALM program One-year prevalence of migraine in Spain: A nationwide population-based survey Cephalalgia 2011 14 463 470 10.1177/0333102410382794 20819843

[B8] Headache Classification Subcommittee of the International Headache Society The International Classification of Headache Disorders, 2nd edition (ICHD-2) Cephalalgia 2004 14 suppl 1 1 160 10.1111/j.1468-2982.2003.00824.x 14979299

[B9] Linde M Stovner LJ Zwart J-A Hagen K Time trends in the prevalence of headache disorders. The Nord-Trøndelag Health Studies (HUNT 2 and HUNT 3) Cephalalgia 2011 14 585 596 10.1177/0333102410391488 21123626

[B10] Lipton RB Bigal ME Diamond M Freitag F Reed ML Stewart WF AMPP Advisory Group Migraine prevalence, disease burden, and the need for preventive therapy Neurology 2007 14 343 349 10.1212/01.wnl.0000252808.97649.21 17261680

[B11] Lipton RB Stewart WF Diamond S Diamond ML Reed M Prevalence and burden of migraine in the United States: data from the American Migraine Study II Headache 2001 14 646 657 10.1046/j.1526-4610.2001.041007646.x 11554952

[B12] Stewart WF Lipton RB Celentano DD Reed ML Prevalence of migraine headache in the United States. Relation to age, income, race, and other sociodemographic factors JAMA 1992 14 64 69 10.1001/jama.1992.03480010072027 1727198

[B13] Hoffmeyer-Zlotnik JHP Developments in applied statistics 2003 Ljubljana: FDV, http://mrvar.fdv.uni-lj.si/pub/mz/mz19/hoff.pdf

[B14] Gosta F Sampling Individuals Within Households in Telephone Surveys 1993 Proceedings of the Survey Research Methods Section of the American Statistical Association, In pp 1113 1118

[B15] Yan T A Meta-analysis of within-household respondent selection methods AAPOR 2009 14 6134 6146

[B16] Deutsches Institut für Normung (DIN) Market, opinion and social research - vocabulary and service requirements (ISO 20252:2006) 2006 Beuth Verlag GmbH, Berlin

[B17] Bortz J Lienert GA Boehnke K Verteilungsfreie Methoden in der Biostatistik. Chapter 5.3.4 1990 Springer, Heidelberg, Berlin

[B18] Goebel H Petersen-Braun M Soyka D The epidemiology of headache in Germany: A nationwide survey of a representative sample on the basis of the headache classification of the International Headache Society Cephalalgia 1994 14 97 106 10.1046/j.1468-2982.1994.1402097.x 8062362

[B19] Lampl C Buzath A Baumhackl U Klingler D One-year prevalence of migraine in Austria: a nation-wide survey Cephalalgia 2003 14 280 286 10.1046/j.1468-2982.2003.00509.x 12716346

[B20] Nikiforow R Headache in a random sample of 200 persons: a clinical study of a population in northern Finland Cephalalgia 1981 14 99 107 10.1111/j.1468-2982.1981.tb00016.x 7346178

[B21] Katsarava Z Dzagnidze A Kukava M Mirvelashvili E Djibuti M Janelidze M Primary headache disorders in the Republic of Georgia: prevalence and risk factors Neurology 2009 14 1796 1803 10.1212/WNL.0b013e3181c34abb 19933983

[B22] Stewart WF Lipton RB Reed ML Medication use and disability among migraineurs: a national probability sample survey Headache 1992 14 5 223 228 10.1111/j.1526-4610.1992.hed3205223.x 1628958

[B23] Stewart WF Lipton RB Liberman J Variation in migraine prevalence by race Neurology 1996 14 52 59 10.1212/WNL.47.1.52 8710124

[B24] Lipton RB Stewart WF Reed M Diamond S Diamond ML Reed ML Migraine's impact today. Burden of illness, patterns of care Postgrad Med 2001 14 1 38 40 43-5 11198257 10.3810/pgm.2001.01.821

[B25] Lipton RB Scher AI Kolodner K Liberman J Steiner TJ Stewart WF Migraine in the United States: epidemiology and patterns of health care use Neurology 2002 14 885 894 10.1212/WNL.58.6.885 11914403

[B26] Henry P Michel P Brochet B Dartigues JF Tison S Salamon R A nationwide survey of migraine in France: prevalence and clinical features in adults Cephalalgia 1992 14 229 237 10.1046/j.1468-2982.1992.1204229.x 1525798

[B27] Henry P Auray JP Gaudin AF Prevalence and clinical characteristics of migraine in France Neurology 2002 14 232 237 10.1212/WNL.59.2.232 12136063

[B28] Hagen K Linde M Steiner TJ Zwart JA Stovner LJ The bidirectional relationship between headache and chronic musculoskeletal complaints: an 11-year follow-up in the Nord-Trøndelag Health Study (HUNT) Eur J Neurol 2012 14 11 1447 1454 10.1111/j.1468-1331.2012.03725.x 22519547

[B29] Stewart WF Simon D Shechter A Lipton RB Population variation in migraine prevalence: a meta-analysis J Clin Epidemiol 1995 14 269 280 10.1016/0895-4356(94)00128-D 7869073

[B30] Bassols A Bosch F Campillo M Canellas M Banos J An epidemiological comparison of pain complaints in the general population of Catalonia [Spain] Pain 1999 14 9 16 10.1016/S0304-3959(99)00069-X 10506667

[B31] Boardman HF Thomas E Croft PR Millson DS Epidemiology of headache in an English district Cephalalgia 2003 14 29 137 10.1046/j.1468-2982.2003.00441.x 12603370

[B32] Fernandez-de-las-Penas C Hernandez-Barrera V Carrasco-Garrido P Alonso-Blanco C Palacios-Cena D Jimenez-Sanchez S Jimenez-Garcıa R Population-based study of migraine in Spanish adults: relation to socio-demographic factors, lifestyle and co-morbidity with other conditions J Headache Pain 2010 14 97 104 10.1007/s10194-009-0176-5 20012124 PMC3452289

[B33] Stang PE Osterhaus JT Impact of migraine in the United States: data from the National Health Interview Survey Headache 1993 14 29 35 10.1111/j.1526-4610.1993.hed3301029.x 8436495

[B34] Carson AL Rose KM Sanford CP Ephross SA Stang PE Hunt KJ Lifetime prevalence of migraine and other headaches lasting 4 or more hours: the Atherosclerosis Risk in Communities (ARIC) study Headache 2004 14 20 28 10.1111/j.1526-4610.2004.04005.x 14979879

[B35] Bigal ME Lipton RB Winner P Reed ML Diamond S Stewart WF AMPP advisory study group Migraine in adolescents: association with socioeconomic status and family history Neurology 2007 14 16 25 10.1212/01.wnl.0000265212.90735.64 17606878

[B36] Pryse-Philhps W Findlay H Tugwell P Edmeads J Murray TJ Nelson RF A Canadian population survey on the clinical, epidemiologic and societal impact of migraine and tension-type headache Can J Neurol Sci 1992 14 333 339 1393842

[B37] Lipton RB Hamelsky SW Stewart WF Silberstein SD, Lipton RB, Dalessio DJ Epidemiology and impact of headache Wolf’s headache and other head pain 2001 Oxford University Press, New York pp 85 107

[B38] Schwartz BS Stewart WF Simon D Lipton RB Epidemiology of tension-type headache JAMA 1998 14 381 383 10.1001/jama.279.5.381 9459472

[B39] Hagen K Zwart J-A Vatten L Stovner LJ Bovim G Prevalence of migraine and nonmigrainous headache-head-HUNT, a large population-based study Cephalalgia 2000 14 900 906 10.1046/j.1468-2982.2000.00145.x 11304025

[B40] Russell MB Kritiansen HA Saltyte-Bentn J Kværner KJ A cross-sectional population-based survey of migraine and headache in 21,177 Norwegians: the Akershus sleep apnea project J Headache Pain 2008 14 339 347 10.1007/s10194-008-0077-z 18850259 PMC3452083

[B41] Sjösten N Nabi H Westerlund H Influence of retirement and work stress on headache prevalence: A longitudinal modelling study from the GAZEL Cohort Study Cephalalgia 2011 14 696 705 10.1177/0333102410394677 21220374 PMC3317892

[B42] Linet MS Wacholder S Zahm SH Interpreting epidemiologic research: lessons from studies of childhood cancer Pediatrics 2003 14 218 232 12837914

[B43] Schaeffer NC Dykema MDW Marsden PV, Wright JD Interviewers and interviewing Handbook of survey research 2010 2 Emerald Group Publishing Ltd, Bingley UK pp 437 470

[B44] Weisberg HF Weisberg HF Survey modes The total survey error approach 2005 University of Chicago Press, Chicago pp 29 42

[B45] Zivadinov R Willheim K Jurjevic A Sepic-Grahovac D Bucuk M Zorzon M Prevalence of migraine in Croatia: a population-based survey Headache 2001 14 805 812 10.1046/j.1526-4610.2001.01147.x 11576206

[B46] Groves RM Fowler FJ Couper MP Lepkowski JM Singer E Tourangeau R Survey methodology – Chapter 5 - Methods of data collection 2004 John Wiley & Sons, Hoboken, New Jersey pp 137 168

[B47] Rasmussen BK Jensen R Olesen J Questionnaire versus clinical interview in the diagnosis of headache Headache 1991 14 290 295 10.1111/j.1526-4610.1991.hed3105290.x 1860786

